# Long-term outcomes after percutaneous withdrawal of HeartWare left ventricular assist device (HVAD) support: A 10-year update

**DOI:** 10.1016/j.jhlto.2024.100169

**Published:** 2024-10-23

**Authors:** Chokanan Thaitirarot, Leonard M. Shapiro, Clive Lewis, Jayan Parameshwar, Steven S.L. Tsui, Stephen J. Pettit

**Affiliations:** a Transplant Unit, Royal Papworth Hospital, Royal Papworth Hospital NHS Foundation Trust, Cambridge, UK; b Cardiology Centre, Chulabhorn Hospital, Chulabhorn Royal Academy, Bangkok, Thailand; c Cardiology Unit, Royal Papworth Hospital, Royal Papworth Hospital NHS Foundation Trust, Cambridge, UK

**Keywords:** cardiac remodeling, heart failure, ventricular assist device, HVAD, decommissioning

## Abstract

Ten years have passed since we reported percutaneous decommissioning of an implantable left ventricular assist device (LVAD) using 2 Amplatzer vascular plugs in a 17-year-old male who was bridged to recovery after 22 months of LVAD support. While his left ventricular (LV) dimensions never completely normalized and there has been persistent mild impairment of LV systolic function, the patient remains free of heart failure symptoms and his natriuretic peptide level has been well suppressed on guideline-directed medical therapy. He is anticoagulated with Warfarin. There have been no adverse events relating to either the decommissioned LVAD or the percutaneous driveline remnant, or anticoagulation. This case highlights the potential for long-term survival without adverse events in individuals who are left with a redundant implantable LVAD after successful percutaneous withdrawal of mechanical circulatory support.

## Background

Left ventricular assist devices (LVADs) have proven effective in improving quality of life and prolonging survival in patients with advanced heart failure.^1^ Adequate recovery of cardiac function to allow for discontinuation of LVAD support is rare, occurring in only 1% of patients during the first 12 months of support.^2^ Traditionally, patients weaned off LVAD support have to undergo an invasive reoperation for explantation of the intracorporeal pump components. This carries considerable risks, specifically pertaining to hemorrhage and myocardial injury during mediastinal dissection.^3^ A perioperative mortality rate within 30 days was reported to be around 10% in a systematic review consisting of 213 patients who underwent LVAD explantation following myocardial recovery.^4^ A less invasive strategy, LVAD decommissioning, involves LVAD deactivation and outflow graft obstruction, which can be achieved via surgical ligation or percutaneous catheter-based approach. This is followed by severing the subcutaneous driveline, removing the percutaneous portion, and leaving the LVAD in situ. The surgical access requires a thoracotomy or subcostal incision and exposes the patients to risks associated with blood transfusion and myocardial trauma during dissection of adhesions.^5^ In light of these considerations, the pursuit of a percutaneous catheter-based withdrawal for LVAD support has emerged as an attractive alternative.

This report serves as an update to our previous case report,^6^ detailing outcomes 1 decade after the minimally invasive percutaneous withdrawal of HeartWare ventricular assist device (HVAD) support in a patient with dilated cardiomyopathy. We highlight the feasibility and safety of this approach.

### Case presentation

We previously reported the case of a 17-year-old male with dilated cardiomyopathy who received a HeartWare HVAD (HeartWare Inc, Framingham, MA). By 22 months of LVAD support, there was significant reverse remodeling with only mild residual left ventricular systolic dysfunction [left ventricular ejection fraction (LVEF) 48% and left ventricular internal diameter in diastole 56 mm], a peak oxygen consumption of 23 ml/min/m^2^, and cardiac index of 3.1 liters/min/m^2^ by thermodilution. LVAD support was weaned and then withdrawn by the placement of 2 vascular plugs (Amplatzer; St. Jude MedicalInc, St. Paul, MN) in the proximal and distal ends of the tubular outflow graft under fluoroscopic guidance. On the following day, the patient was taken to the operating room for excision of the percutaneous section of the driveline. Both the HVAD pump and the occluded outflow graft were left in situ.

### Clinical course and management

**Figure 1:**
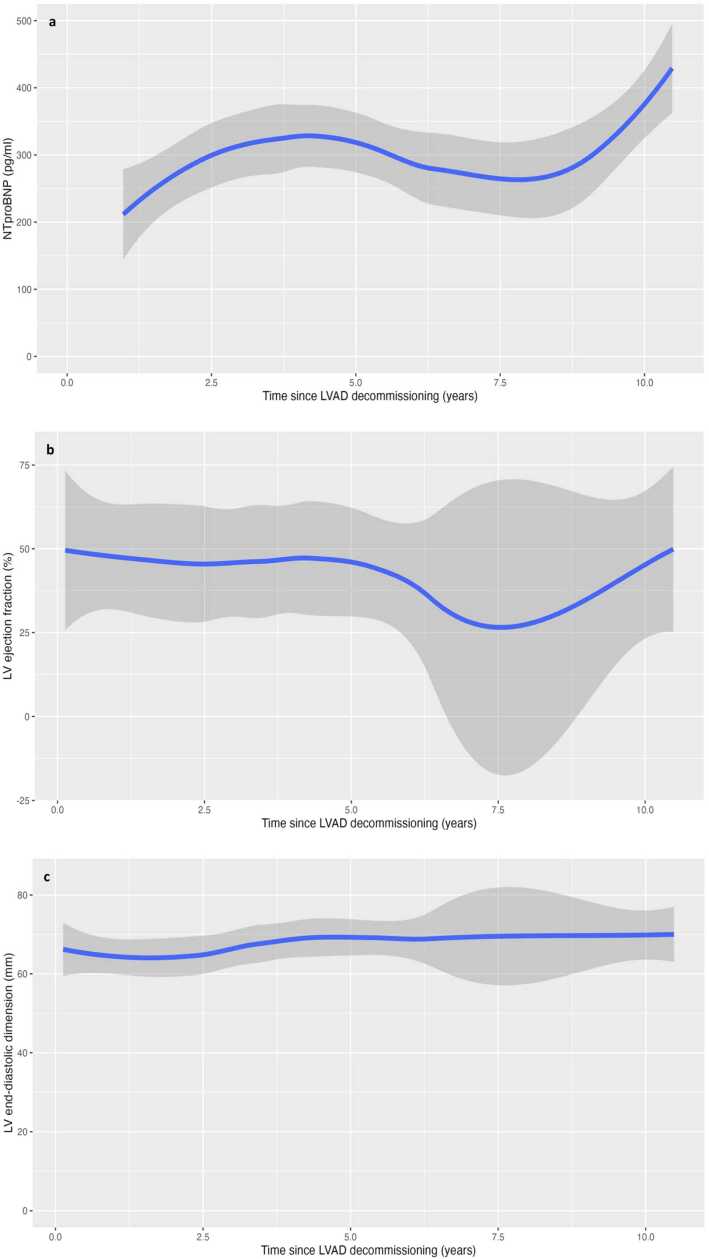
(a) N-terminal pro-brain natriuretic peptide (NT-proBNP) levels, (b) left ventricular internal dimension at end-diastole, and (c) left ventricular ejection fraction (LVEF) over the years following the left ventricular assist device (LVAD) decommissioning. A smoothed conditional mean using the loess technique (blue line) and standard error (gray area) are presented. LV, left ventricular.

The patient was initially treated with guideline-directed medical therapy including Ramipril, Carvedilol, and Spironolactone. Outcome data were analyzed in R version 4.4.1 (R Foundation for Statistical Computing) using the package ggplot2 version 3.5.1. N-terminal pro-brain natriuretic peptide (NT-proBNP) levels have consistently remained below 500 pg/ml (Figure 1a). Six years post decommissioning, his LVEF decreased from 45% to 36% on a serial transthoracic echocardiogram, despite the absence of heart failure symptoms. Subsequent conversion from Ramipril to Sacubitril-Valsartan and introduction of Dapagliflozin led to a significant improvement in the LVEF, rising to 50%. A decade following the decommissioning of the LVAD, the patient has remained asymptomatic and consistently in New York Heart Association Class I. Changes in left ventricular internal diameter in diastole and LVEF over time are illustrated in Figure 1b and c, respectively.

**Figure 2:**
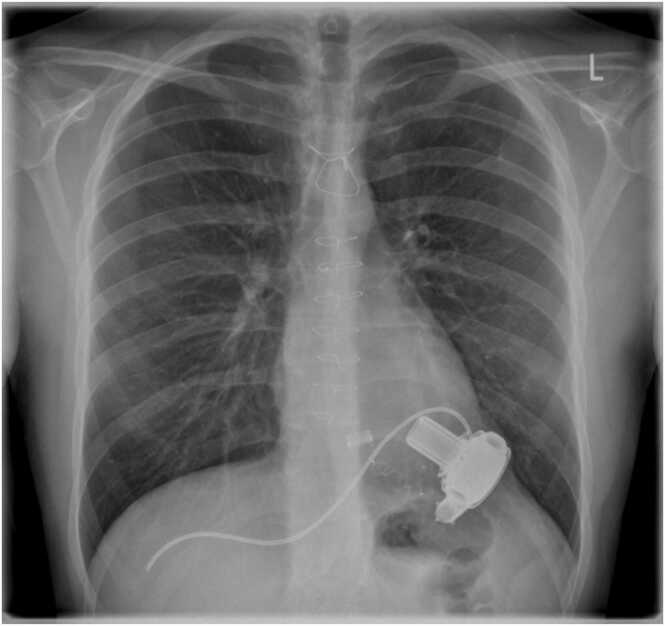
The most recent chest X-ray (CXR) showing a normal cardiothoracic ratio with the presence of the decommissioned HeartWare HVAD.

**Figure 3:**
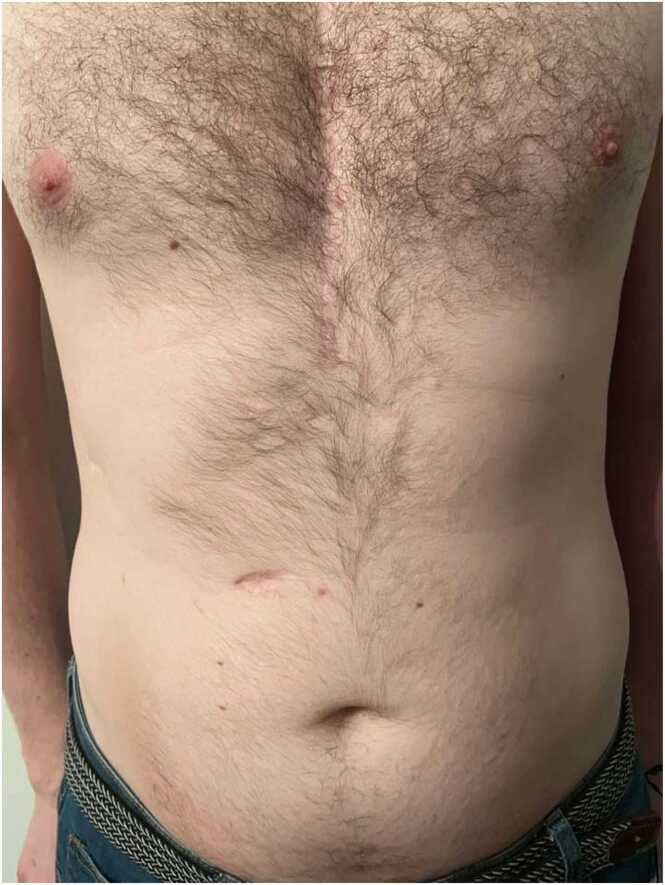
A current photograph of the patient’s abdomen a decade following the left ventricular assist device (LVAD) decommissioning.

He continues to be anticoagulated with Warfarin targeting an international normalized ratio of 2 and 3. Throughout this period, the patient has not experienced any adverse events related to the decommissioned HVAD. This includes the absence of infection, thrombo-embolic events, or any hardware-related complications. There have been no hospitalizations for heart failure. The most recent chest X-ray (CXR) showed a normal cardiothoracic ratio with the presence of the decommissioned HVAD (Figure 2). Given the absence of complications, there are no plans to remove the deactivated HVAD, unless a complication arises due to the redundant system or anticoagulation. A current photograph of the patient’s abdomen is shown in Figure 3.

## Discussion

This case exemplifies the possibility of a long-term, complication-free course following the minimally invasive percutaneous withdrawal of LVAD support. The decommissioned device has remained inert without contributing to any adverse clinical outcomes over a decade. There have been several case series and reports of successful percutaneous LVAD decommissioning, with follow-up periods extending up to 3.5 years.^7^ Long-term outcomes are rarely reported. Various devices have been utilized for LVAD decommissioning, with the Amplatzer vascular plug emerging as the most common choice.^7^

The most recent systematic review comparing patient outcomes following LVAD withdrawal via minimally invasive LVAD decommissioning vs explantation provided valuable insights.^8^ Among 85 patients from 44 studies, 17 underwent withdrawal by decommission, with only 7 of these patients receiving percutaneous occlusion of the outflow graft using a vascular plug.^8^ At a median follow-up of 389 days, no significant differences were noted in the incidence of cerebrovascular accidents, infection, or survival, although there was a trend toward a higher risk of heart failure recurrence in patients undergoing decommission.^8^ Long-term follow-up involving more patients is required to evaluate the optimal treatment course in these patients. Furthermore, it is imperative to acknowledge the paucity of comparative data in the current literature on perioperative mortality rates between LVAD decommission and explantation, warranting further investigation.

LVAD decommissioning offers several advantages over LVAD explantation, including the avoidance of major surgery and the potential to maintain the apical orifice should subsequent LVAD reimplantation become indicated.^9^ These factors make transcatheter LVAD decommissioning an attractive option, particularly for patients deemed at high risk of heart failure recurrence and/or for device explantation. However, the decision to leave the device in place necessitates consideration of potential risks, such as systemic thromboembolism, the requirement for continued anticoagulation, and the possibility of infection. Fortunately for this patient, these had not been encountered. Further studies are needed to evaluate these potential long-term complications.

## Conclusion

In conclusion, we have described long-term outcomes of a case with percutaneous withdrawal of HeartWare LVAD. Our patient's decade-long journey post decommission offers an insight into the long-term safety and feasibility of this approach.

## Author contribution

C.T., S.T., and S.P. conceived the idea of the study. S.P. supervised the study, providing guidance and support throughout. L.S., C.L., J.P., S.T., and S.P. were involved in the clinical care of the patient. C.T. wrote the first draft of the article with support from S.P.; L.S., C.L., J.P., S.T., and S.P. reviewed the article before submission. All authors have approved the final draft of the article.

## Patient consent for publication

Written consent was obtained.

## Disclosure statement

S.T. reports consulting fees from 3R Life Sciences Ltd.

Funding and Acknowledgments: None.

## Data Availability

Data are available upon reasonable request from the author at chokanan.thaitirarot@nhs.net.
